# Pore-scale imaging and analysis of low salinity waterflooding in a heterogeneous carbonate rock at reservoir conditions

**DOI:** 10.1038/s41598-021-94103-w

**Published:** 2021-07-23

**Authors:** Ahmed M. Selem, Nicolas Agenet, Ying Gao, Ali Q. Raeini, Martin J. Blunt, Branko Bijeljic

**Affiliations:** 1 Department of Earth Science and Engineering, Imperial College London, London, SW7 2AZ UK; 2 TOTAL E&P, Centre Scientifique et Technique Jean Féger (CSTJF), 64000 Pau, France

**Keywords:** Crude oil, Chemical engineering, Fluid dynamics, Energy science and technology, Chemistry

## Abstract

X-ray micro-tomography combined with a high-pressure high-temperature flow apparatus and advanced image analysis techniques were used to image and study fluid distribution, wetting states and oil recovery during low salinity waterflooding (LSW) in a complex carbonate rock at subsurface conditions. The sample, aged with crude oil, was flooded with low salinity brine with a series of increasing flow rates, eventually recovering 85% of the oil initially in place in the resolved porosity. The pore and throat occupancy analysis revealed a change in fluid distribution in the pore space for different injection rates. Low salinity brine initially invaded large pores, consistent with displacement in an oil-wet rock. However, as more brine was injected, a redistribution of fluids was observed; smaller pores and throats were invaded by brine and the displaced oil moved into larger pore elements. Furthermore, in situ contact angles and curvatures of oil–brine interfaces were measured to characterize wettability changes within the pore space and calculate capillary pressure. Contact angles, mean curvatures and capillary pressures all showed a shift from weakly oil-wet towards a mixed-wet state as more pore volumes of low salinity brine were injected into the sample. Overall, this study establishes a methodology to characterize and quantify wettability changes at the pore scale which appears to be the dominant mechanism for oil recovery by LSW.

## Introduction

Carbonate reservoirs are critical for energy supply as they host more than half of the world’s remaining conventional oil reserves as well as large amounts of natural gas^1^. However, producing carbonate reservoirs can be challenging due to the intricate fluid flow and the retention of oil to formation surfaces under oil-wet conditions. Waterflooding is one of the main techniques that has been frequently implemented to improve production by maintaining pressure and displacing oil^2^. The water used in this method is typically a highly saline solution which is either sea water or produced water that is separated from the oil and re-injected back into the reservoir. Injecting a high salinity brine may leave behind a significant remaining oil saturation^3^. The results from previous studies, and some field trials, indicate that a further enhancement in sweep efficiency and oil recovery can be achieved when the salinity and/or composition of the injected brine is changed^4^. Injecting brine with reduced salinity and modified ionic composition into oil reservoirs is known as low salinity waterflooding (LSW) and is a promising enhanced oil recovery (EOR) technique due to its relatively low cost and minimal environmental impact^5^.

From previous experimental work, it is understood that low salinity brine can cause changes induced by physico-chemical mechanisms in both sandstone^6–12^ and carbonate rocks^13–19^ resulting in an incremental increase in oil recovery. Some field trials have supported these findings^3, 9, 20–22^. Wettability alteration towards increased water-wetness is the most frequently suggested cause of this increased recovery^23–27^. Despite considerable efforts to explain its origin and underlying mechanisms, pore-scale LSW processes remain poorly understood^28^. Until recently, numerous studies investigating wettability measurements at the core scale were conducted from which it was not possible to assert how the putative mechanisms are responsible for the low salinity effect (LSE) at the pore scale^28, 29^. Therefore, addressing the connection between the low salinity-induced wettability changes and incremental oil recovery across different length scales is fundamental to further understand LSW as an EOR technique^29^.

Recent advances in X-ray microtomography (micro-CT) have made it possible to image fluids within the rock pore space at micron resolution in a non-destructive manner^30^. This technique can be deployed for different applications including improved oil recovery and carbon dioxide storage^31^. Combining this non-invasive technique with a high-pressure and high-temperature apparatus has enabled the study of multiphase flow in representative geological systems under subsurface conditions^32–34^. This method has been previously deployed to characterize rock wettability, fluid displacement patterns and relative permeability^35–38^. Few studies have used x-ray imaging to investigate LSW^39–44^. However, there is a lack of experimental work performed on heterogeneous carbonate rocks at reservoir conditions using reservoir fluids. Additionally, there are some limitations in previous studies with regards to the wettability characterization analyses. This includes investigating fluid configurations on small sections of the studied samples, and using a manual method to obtain only a few contact angle measurements.

The aim of this study is, therefore, to gain unprecedented insights into LSW by applying several methods which combine coreflooding, X-ray imaging and image analysis to investigate the pore-scale effects of LSW on a complex crude oil/brine/rock system. To this end, the carbonate rock sample was exposed to a constant flow of crude oil under high temperature to achieve an oil-wet state. Low salinity brine was then injected at different flow rates corresponding to capillary numbers ranging from 10^–8^ to 10^–7^. Then high resolution micro-CT images were acquired to capture the fluid saturation and spatial distribution as well as to assess the wetting state of the system at different stages during the waterflood. This study is designed to shed light on the relationship between wettability changes induced by LSW and flow rate, contact angles and curvatures (from which capillary pressure can be found), fluid distribution and recovery to further understand how LSW can be employed to enhance oil recovery from carbonate reservoirs.

## Materials and methods

### Rock and fluid properties

A cylindrical sample, 5.9 mm in diameter and 10 mm in length, was used in this study. The rock was an Estaillades limestone, quarried in France, composed of 97 weight% calcite with traces of dolomite having a bimodal pore structure^45, 46^. Our sample had a porosity of 0.293 ± 0.003, measured using a helium porosimeter. The absolute permeability of the sample was calculated by measuring the pressure drop during brine injection and, by applying Darcy’s law: a value of 0.11 ± 0.03 Darcy was calculated. Formation brine and low salinity brine were prepared in the lab as synthetic solutions of various salts in deionized water, Table 1, using salts with a grade above 99.5%. Formation brine was prepared to a similar composition as the brine in the reservoir from which the crude used in this study was extracted. The crude oil was from a producing oil field and its composition is also shown in Table 1. It has a density of 803 kg/m^3^ and a viscosity of 1.56 mPa s measured at 70 °C (the temperature conditions for the waterflood experiments). The viscosity of the low salinity brine is 0.426 mPa s, and the interfacial tension between oil and low salinity brine is 26.3 mN/m, both measured at 70 °C.

**Table 1:** Composition and properties of the fluids used in this study.

Brine	Formation brine (g/L)	Low salinity brine (g/L)
**Salt**		
NaCl	109.55	0.76
KCl	0	0.035
CaCl_2_·2 H_2_O	46.07	0.02
MgCl_2_·6 H_2_O	11.24	0.296
Na_2_SO_4_	0.14	0.087
NaHCO_3_	0.2	0.0007
**Crude oil**		
Total acid number (mg KOH/g)		0.34
Total base number (mg KOH/g)		0.41
Saturates (wt%)		48.4
Aromatics (wt%)		48.1
Resins (wt%)		2.8
Asphaltenes (wt%)		0.7

### Flow apparatus and experimental procedure

The experimental apparatus is made up of an X-ray transparent Hassler type carbon fibre flow cell connected to syringe pumps to apply confining and line pressures and control the fluid flow rate inside the sample (Fig. S1). This study was performed in three stages consisting of: (i) preparing and saturating the sample with formation brine, (ii) injecting crude oil over 3 weeks at high pressure and temperature to change rock wettability, and (iii) studying the effect of injecting low salinity brine at different flow rates by acquiring X-ray images using the micro-CT scanner.

The sample was initially flooded with brine solution made from deionized water with 20 weight% potassium iodide (KI) to characterize the rock bimodal porosity. The sample was then cleaned, dried and vacuumed. Formation brine was injected to fully saturate the rock sample. For drainage, we used a synthetic viscous oil to reach a low initial water saturation in the sample. This was followed by injecting 10 pore volumes of toluene to avoid any mixing between the synthetic and crude oil. To change the rock wettability to a similar state found in oil reservoirs, the core underwent a process called ageing which involved continuous injection of crude oil for 3 weeks at a low flow rate (2 µL/min) at high pressure (11 MPa) and temperature (80 °C). The flow direction was reversed at the mid-point during injection to ensure a uniform distribution of oil, and wettability alteration, across the sample. After ageing, the crude oil in the sample was replaced by doped crude oil (20 weight% 1-iododecane) to allow for sufficient contrast in the X-ray images between oil and brine and a scan was acquired to assess the rock wettability state. We assume that replacement of the crude oil does not affect the wetting state^39^.

### Waterflooding

Low salinity brine was injected in secondary mode, i.e. at irreducible formation water saturation, into the predominantly oil-saturated rock sample at a sequence of increasing flow rates; 1, 2, 4, 11, 22 and 42 µL/min at 70 °C. The capillary number associated with each flow rate was calculated as

1 $$\begin{array}{*{20}c} {Ca = \frac{{{\upmu {\text{q}}}}}{\sigma } } \\ \end{array}$$

where σ is the oil–brine interfacial tension, μ is the viscosity of brine and q is the Darcy velocity of brine. The calculated values of the capillary number during flooding are shown in Table S1 of the Supplementary Information and range from 9.72 × 10^–9^ to 4.08 × 10^–7^ indicating that we remained in the capillary dominated regime for the entire experiment.

### Image acquisition, processing and segmentation

A Thermo Fisher Heliscan micro-CT was used to acquire high-resolution three-dimensional (3D) images to characterize pore-scale low salinity processes. A tomogram of the rock sample used in this study is shown in Fig. S2 in the Supplementary Material. X-ray images were acquired at different stages: before fluid injection (dry image), after KI-brine injection, after ageing and during LSW. After waiting for 2 hours for stabilization at the end of each low salinity brine injection step, a micro-CT image of the whole sample was acquired, except for the 22 µL/min step. Each acquisition took 20 h and the size of the acquired images was 2800 × 2800 × 3680 voxels, with a voxel size of 2.9 µm. The images were then registered to the reference (dry) image and filtered using non-local means edge-preserving filter to remove image noise^47^. All images were segmented into different phases using a seeded watershed algorithm^48, 49^, as shown in Fig. S3. Fluid saturation was quantified from the segmented images using the volume of fluids in the resolved (macro-) pore space, Fig. S4. More details on image acquisition, processing and segmentation are provided in the Supplementary Information.

### Porosity characterization

We assessed the rock dual-porosity (that is macro pores that can be explicitly resolved in the image and micro-porosity below the image resolution) using the differential imaging technique^50^ described in the pore space characterization section in the Supplementary Information. Our analysis showed that the rock sample has 0.128 and 0.171 macro- and micro-porosity, respectively: a total of 0.299 ± 0.007. which agrees, within experimental uncertainty, with the total helium-measured porosity of 0.293 ± 0.003. Based on observations from the X-ray images acquired, we assumed that the micro-porosity remained water-saturated. The greyscale value of the microporous grains did not change when doped oil was introduced nor during waterflooding indicating that these sub-resolution pores were brine-saturated throughout the experiment. Since micro-pores are below the resolution of the micro-CT scanner (3 µm/voxel), further analysis will focus on macro-pores where fluid saturation, as well as contact angles and curvatures between the different phases, can be measured. The majority of resolved pore elements have radii in the range of 15 to 60 µm for pores and 5 to 30 µm for throats.

### Wettability characterization

Several methods were applied to identify the pore-scale low salinity processes and the wettability state of the rock before and after LSW. Fluid occupancy in the pore space was investigated by a generalized pore network extraction algorithm^51, 52^ using segmented 3D images. This algorithm subdivides the macroscopic void space into pores (a wide region of the pore space) bounded by throats (a restriction in the pore space). The radius of a pore or throat is identified as the radius of the largest sphere that can fit in the void space in a wide region, or restriction respectively, without crossing the solid. Each voxel in the void space identified from segmented 3D images is assigned to a certain pore or throat. The volume of the pore and throat voxels and their inscribed radii were used to analyse the statistics of the pore and throat fluid occupancy. To quantify fluid occupancy, the fraction of oil-filled voxels in the largest sphere was calculated. A pore element is defined to be filled by a certain fluid if the fraction of this fluid is more than a half.

An automated algorithm was used to identify the contact angle distribution^53^. This method allows for hundreds of thousands of in situ contact angle measurements to be performed which cannot be achieved manually^54^. This approach finds the contact angle between immiscible fluids and the rock surface by defining perpendicular vectors to these surfaces, fitting smoothed curvature surfaces to the fluid/fluid interfaces, and recording their intersection with the rock surface.

Curvatures were obtained by approximating the oil–brine interface locally as a quadratic form. The eigenvalues and eigenvectors of this quadratic form represent the principal curvature values and directions, respectively^55–57^. More details on pore occupancy, contact angle and curvature analysis are provided in the Image analysis section in the Supplementary Information.

## Results and discussion

The segmented three-dimensional pore-scale images were used to quantify fluid saturation and oil recovery and to perform pore-by-pore occupancy analysis after low salinity waterflooding. To assess the impact of the low salinity effect on rock wettability with increasing brine injection rate, we measured contact angles and curvatures on oil–brine interfaces extracted from the micro-CT images and calculated capillary pressure.

### Fluid distribution and oil recovery

**Figure 1:**
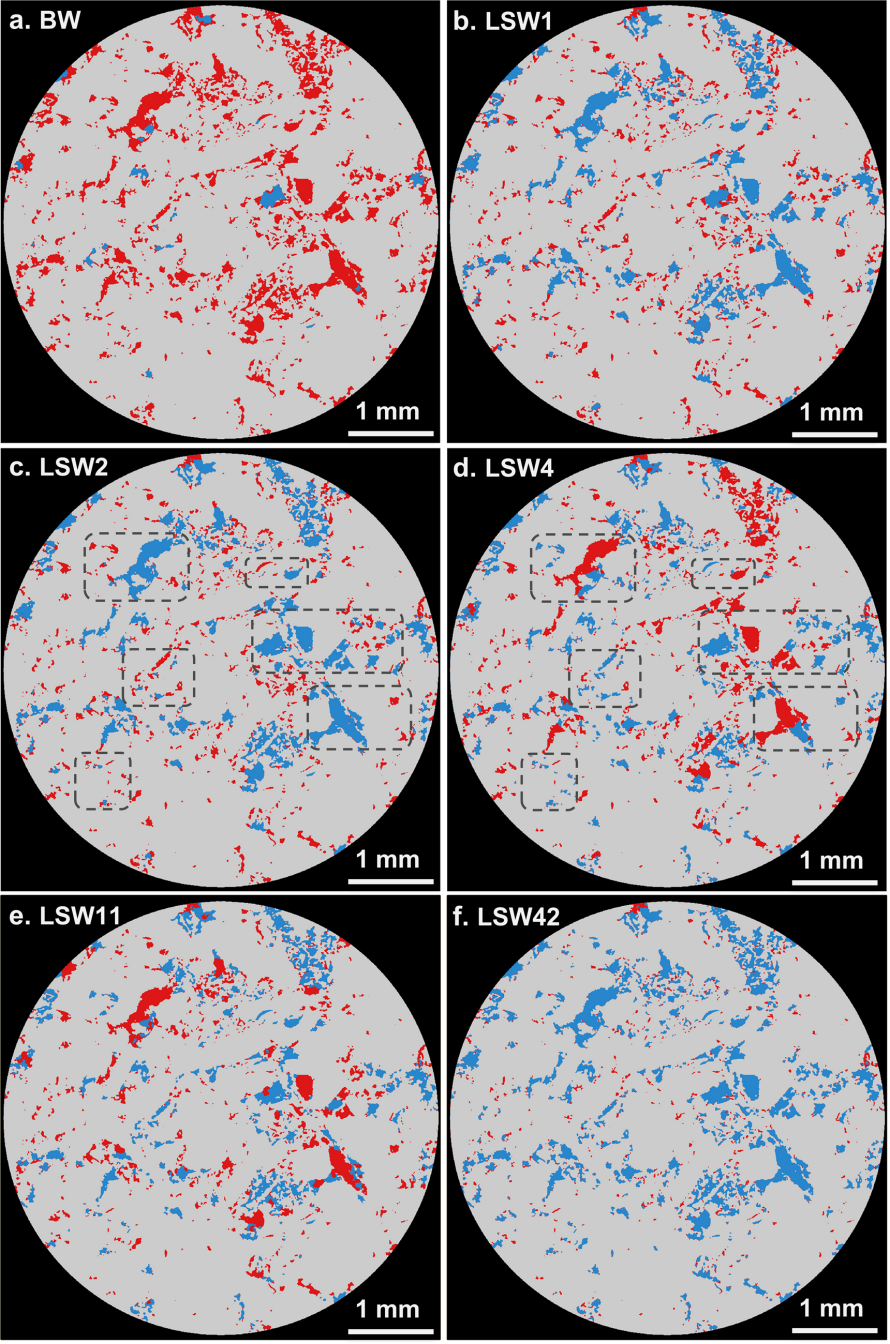
Two dimensional cross-sectional views of the segmented three-dimensional images of oil (red), brine (blue) and rock phase (grey). (**a**) Before waterflooding (BW) oil occupied most of the pore space with a saturation of 88%. (**b,c**) After the first two floods, at 1 and 2 µL/min, low salinity brine mainly invaded larger pores. (**d,e**) After the intermediate floods, 4 and 11 µL/min, fluid redistribution was observed. The highlighted areas show displacement of oil from small pores, with larger pores reoccupied by oil as more pore volumes of brine were injected at higher flow rates. (**f**) After the last flood, 42 µL/min, brine displaced most of the remaining oil. The 3D pore-scale images were segmented and visualized using Avizo 9.5 sofware (https://www.fei.com/software/amira-avizo/).

At the end of ageing, the value of irreducible water saturation in macro-pores measured from the segmented image was 12% as oil occupied most of the pore space (Fig. 1a). As mentioned previously, low salinity brine was then injected at six different flow rates (10 pore volumes at each rate) under capillary-force dominated conditions.

At the early stages of waterflooding, brine preferentially invaded larger pores whereas smaller pores remained uninvaded (Fig. 1b,c): this is expected for an oil-wet rock, where water displaces oil in the larger oil-wet pores through forced injection^58^. After the third injected flow rate (4 µL/min), a different behaviour was observed, where brine started to invade smaller pores and oil re-emerged in larger pores. Remember that we only consider the macro pores resolvable in the image in this analysis. This is consistent with a change in wettability to be more water-wet: brine now preferentially invades the smaller water-wet portions of the pore space, whereas the displaced oil occupies the larger pores. This can be seen when comparing the highlighted areas in Fig. 1c,d. This pattern continued into the next injection step (11 µL/min), with oil and brine occupying both smaller and larger pores (Fig. 1e); however, as the waterflood rate further increased brine displaced most of the oil (Fig. 1f).

**Figure 2:**
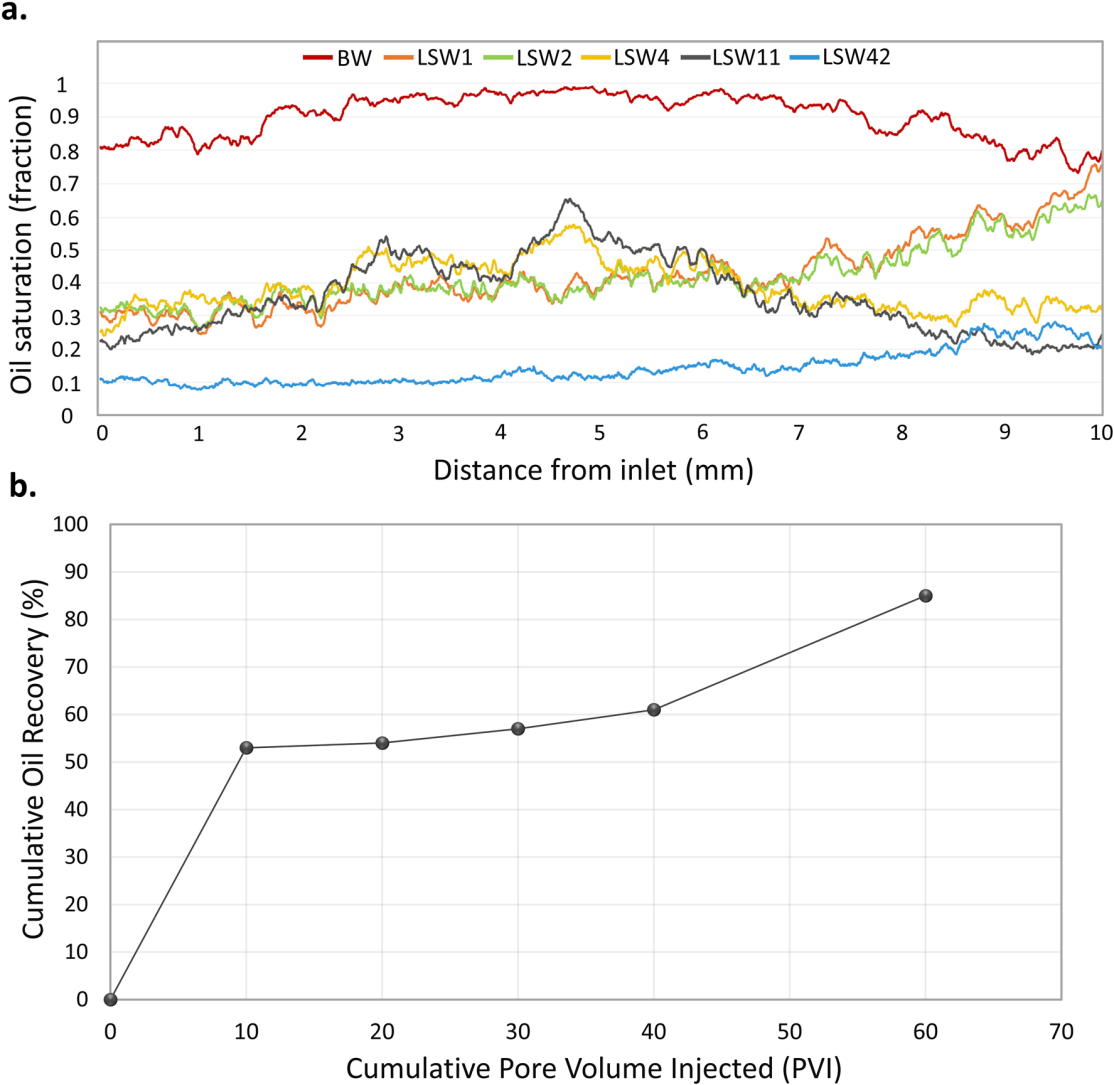
(**a**) Oil saturation profiles across the sample (flow direction from left to right) before waterflooding (BW) and at different stages of LSW, namely 1, 2, 4, 11 and 42 µL/min. (**b**) Oil recovery as a percentage of the oil initially in place in macro-pores. A total of 60 pore volumes (10 at each flow rate) of low salinity brine were injected.

To further examine the effect of LSW across the whole sample and quantify oil recovery, saturation values were determined from the segmented images. After the first two injection rates, more than half of the volume of oil has been displaced across the sample except from the part near the outlet where there was a higher oil saturation, Fig. 2a. Low salinity brine may not have fully displaced formation brine to come in contact with oil and alter wettability in this region of the sample, explaining the high remaining oil saturation. As more low salinity brine was injected at higher rates, 4 and 11 µL/min, a significant amount of oil was displaced from this region near the outlet; however, an increase in oil saturation was observed in the middle part of the sample. This is due to the displacement of oil from smaller into larger pore elements controlled by local wettability and capillary pressure, as shown previously in the segmented 3D images in Fig. 1. This will be discussed further in the pore occupancy analysis in the next section. Two bump rates, 22 and 42 µL/min were then applied to remove any capillary end effects and appropriately assess the remaining oil saturation. At the end of waterflooding, a more uniform saturation profile was achieved with an ultimate recovery value of 85% of the oil initially in place in macro-pores, see Fig. 2b.

### Fluid occupancy mapping

**Figure 3:**
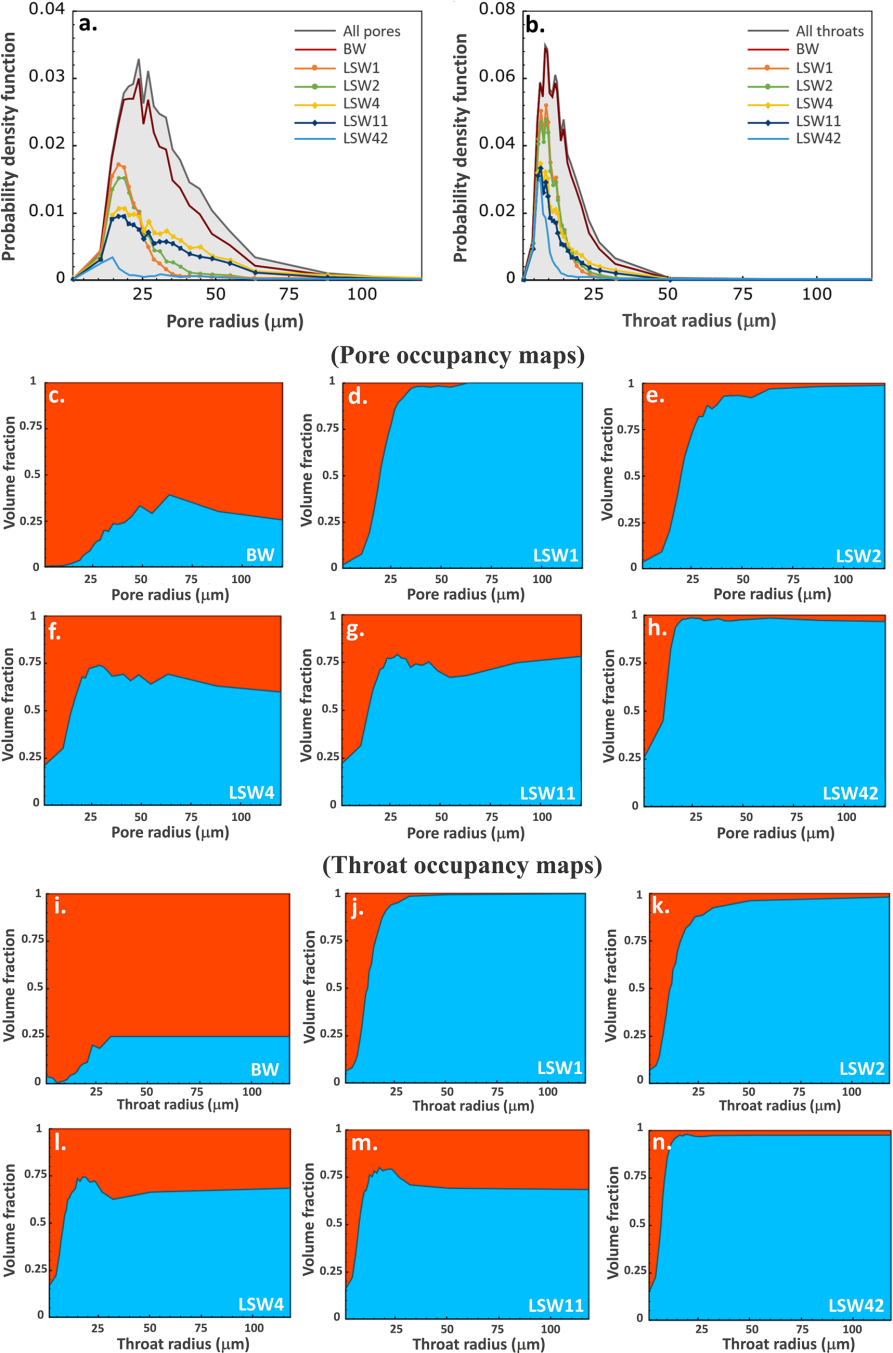
(**a,b**) Histogram plots showing the distribution of oil-filled pore elements at different stages of waterflooding. (**c–n**) Pore and throat occupancy maps showing the volume fraction of pores and throats of different radii occupied by oil (red) and brine (blue).

Mapping of pore-scale fluid occupancy is essential to build a better understanding of the fluid configuration in the pore space and to test whether there were substantial changes in both pore and throat occupancy due to low salinity injection. The size distribution of resolved pores and throats and the volume-weighted fraction of oil-filled pore elements computed using the generalized pore network extraction tool are shown in Fig. 3. The pore radii are mainly between 15 and 60 µm (Fig. 3a), and between 5 and 30 µm for throats (Fig. 3b). Before waterflooding, oil occupied almost all the pore space with brine residing mainly in larger pores (Fig. 3a,c) and throats (Fig. 3b,i). This fluid distribution is characteristic of oil-wet conditions^59, 60^. After the first two waterfloods, brine displaced oil from large and medium-sized pores (pore radius > 20 µm) whereas most of the small pores remained filled with oil, Fig. 3d,e.

As more brine was injected at higher rates (4 and 11 µL/min; LSW4 and LSW11), there was no significant increase in recovery, Fig. 2b (between 30 and 40 pore volumes injected), but brine invaded some smaller pores and oil occupied larger pores, Fig. 3f,g. This redistribution has not been captured in previous studies^42^ and is consistent with a shift towards more water-wet conditions. This fluid configuration where both oil and brine fill large and small pores is consistent with a mixed-wet state with both oil-wet and water-wet regions of the rock^6, 36, 61, 62^: brine preferentially displaced oil from the smaller water-wet elements, while oil filled larger pores. In regions of the pore space that remained oil-wet, oil was not displaced until the flow rate was increased. After the bump rates, brine had invaded the majority of the pores, with some oil remaining in small and large pores, Fig. 3h. A similar tendency of brine filling and oil redistribution was observed in throats, see Fig. 3i–n. The brine filling sequence and oil rearrangement observed during waterflooding are indicative of alteration of the rock wettability by LSW from oil-wetting to a mixed-wetting state.

In the next section, we further investigate this understanding through the analysis of contact angle and curvature measurements to assess the magnitude of wettability alteration during low salinity waterflooding.

### Contact angles, curvatures, and capillary pressure

**Figure 4:**
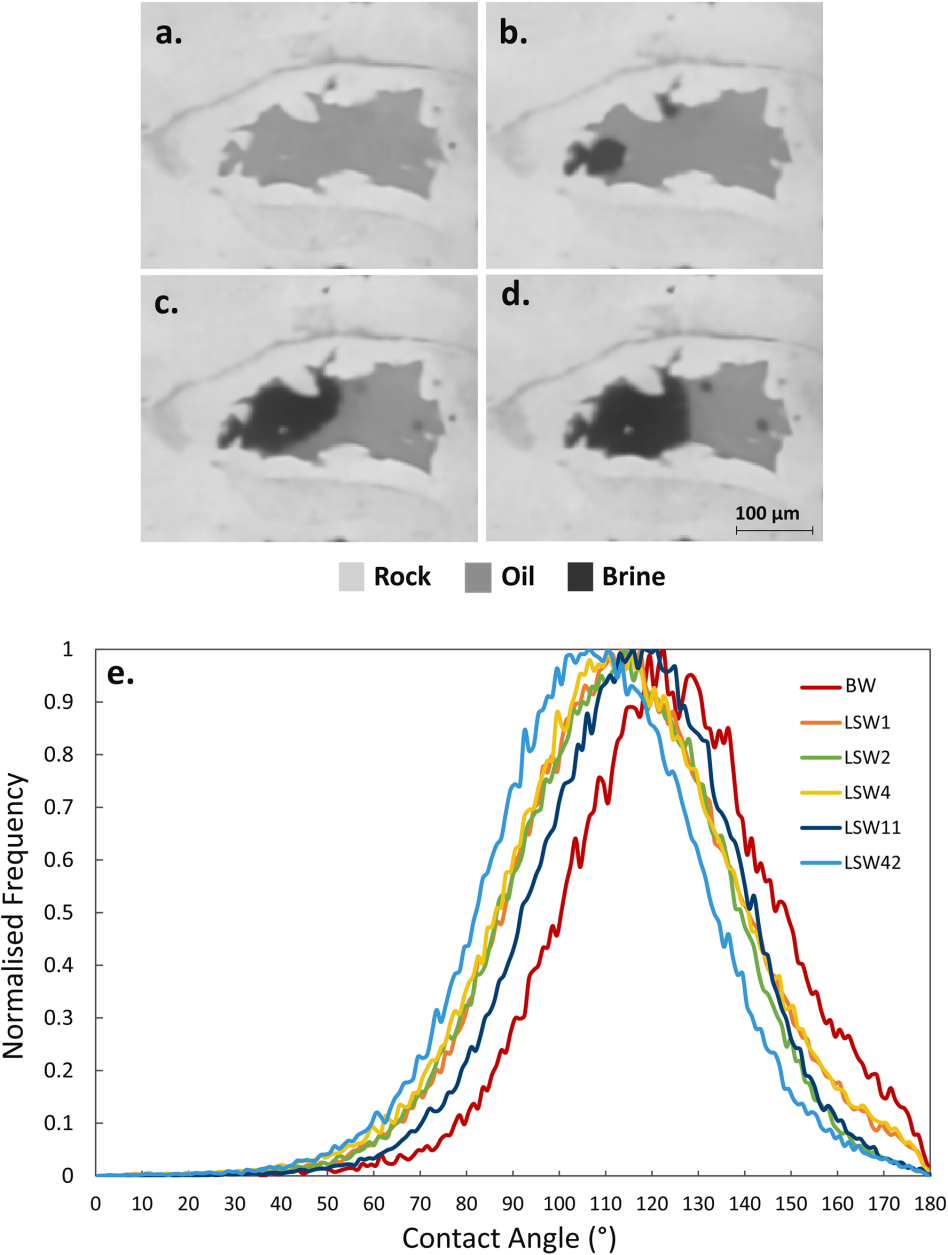
(**a–d**) Two-dimensional raw images illustrating the changes in contact between the fluids and rock in an oil-wet pore before waterflooding (**a**) and after an injection of 20, 30 and 40 pore volumes of low salinity water (**b–d**) respectively. (**e**) Histograms of the in situ contact angle distributions measured on a sub-volume extracted from the segmented 3D images. The 3D pore-scale images were acquired by X-ray micro-tomography with a voxel size of 2.9 µm, and visualized using Avizo 9.5 sofware (https://www.fei.com/software/amira-avizo/)**.**

Contact angle and curvature measurements were performed to quantify wettability changes in large 3D volumes of the complex pore space. Wettability can be inferred from the estimated spatial distribution of contact angles between the fluids within the pore space, also known as the geometric contact angle^48, 63^. An illustration of the contacts between oil, brine and rock, shown in Fig. 4a–d, shows that the brine was observed to be initially bulging into oil in an oil-wet pore. However, with the progress of the waterflood, the oil–brine interface gradually changed to eventually indicate a contact angle close to 90°, reflecting an alteration in the local wetting state. We hypothesize that this is the origin of the increase in recovery, since the flow rates remain in the capillary-dominated regime.

The distribution of in situ contact angles between oil and water was measured throughout the displacement using the automated method on a sub-volume of size 1.5 × 1.5 × 1.5 mm^3^. This sub-volume is located in the top half of the sample, Fig. S2. The contact angle distributions are shown in Fig. 4e. We observe that the mean contact angle before waterflooding, based on over 50,000 measurements, has a value of 124 ± 23°, indicating oil-wet conditions and confirming the success of the ageing process. The contact angle distributions after the brine injection steps show a shift towards water-wetness and a decrease in the mean contact angles. This agrees with the oil redistribution observed from the pore occupancy analysis, Fig. 3. At the end of waterflooding, a further shift in the measured contact angles distribution was observed with a mean value of 108 ± 23°, based on over 100,000 measurements, see Table S2. This change in the contact angle distributions and mean values suggest that the rock, under the effect of low salinity, now displays a mix of water-wet and oil-wet surfaces^54^.

**Figure 5:**
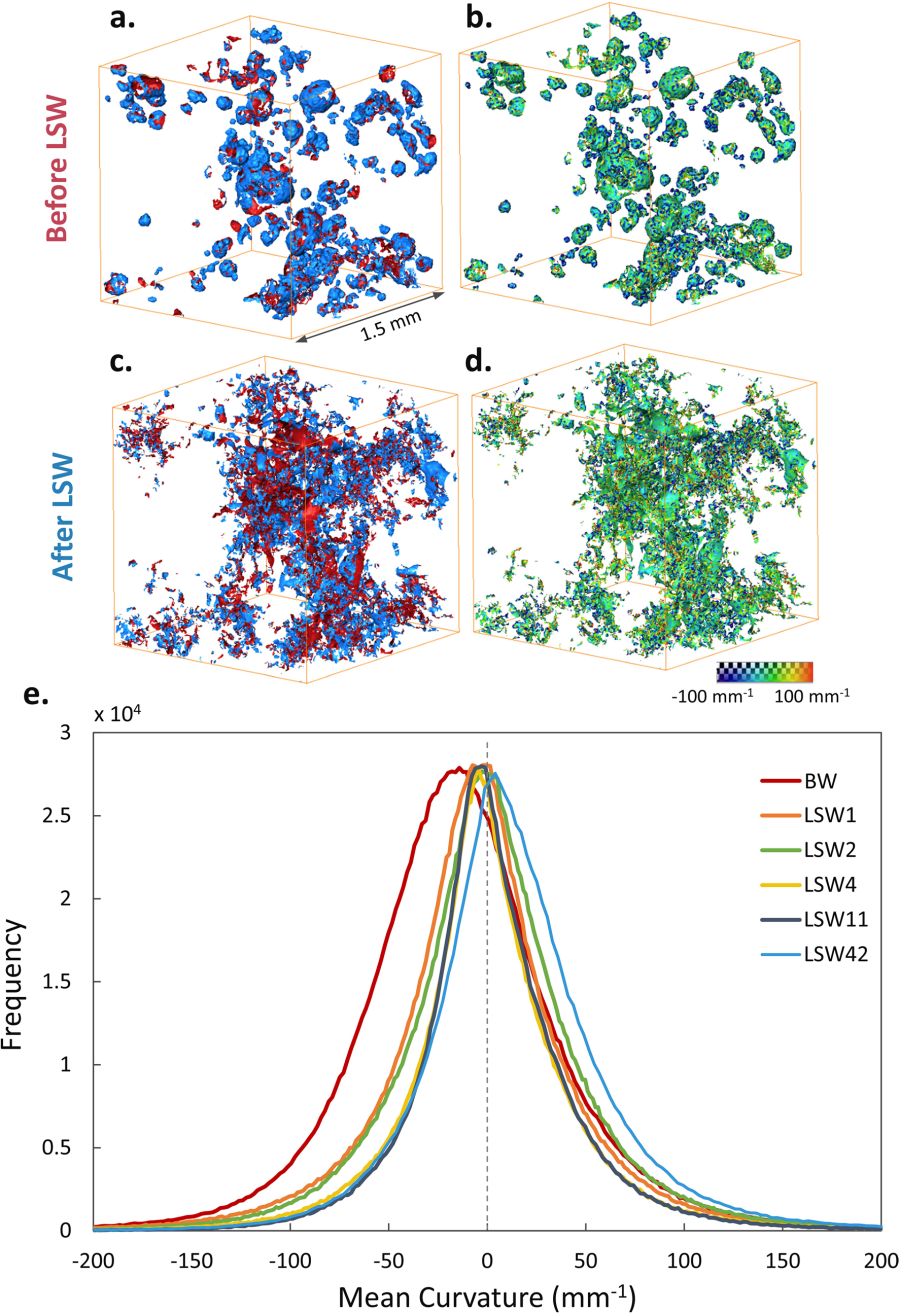
Three-dimensional view of smoothed oil–brine interfaces, from a 1.5 mm^3^ sub-volume, before and after waterflooding (**a,c**) and their measured mean curvatures (**b,d**). Histograms of the mean curvature values measured before and throughout waterflooding (**e**). The interfaces and mean curvatures were extracted from 3D X-ray images using Avizo 9.5 sofware (https://www.fei.com/software/amira-avizo/)**.**

Curvatures of the oil–brine interfaces were captured to reveal details about rock wettability and fluid connectivity as well as to calculate local capillary pressures. Oil–brine interfaces were extracted from the segmented images and smoothed to measure mean curvature values, Fig. S8. The mean curvature κ was calculated as the average of the two principal curvatures (κ_1_ and κ_2_) obtained from the smoothed interfaces, see Fig. 5. Before waterflooding, the principal curvatures are mainly negative resulting in negative mean curvature values. This is consistent with the morphology of the fluid interfaces with water bulging into oil forming ball-shaped structures, see Fig. 5a,b. At the end of waterflooding, there is a mixture of both positive and negative curvatures with roughly equal fractions, and hence a less negative mean curvature value, as shown in Fig. 5c,d. The distributions, in Fig. 5e, show a shift in the mean curvature value from a negative value before waterflooding into values close to zero after low salinity injection.

**Figure 6:**
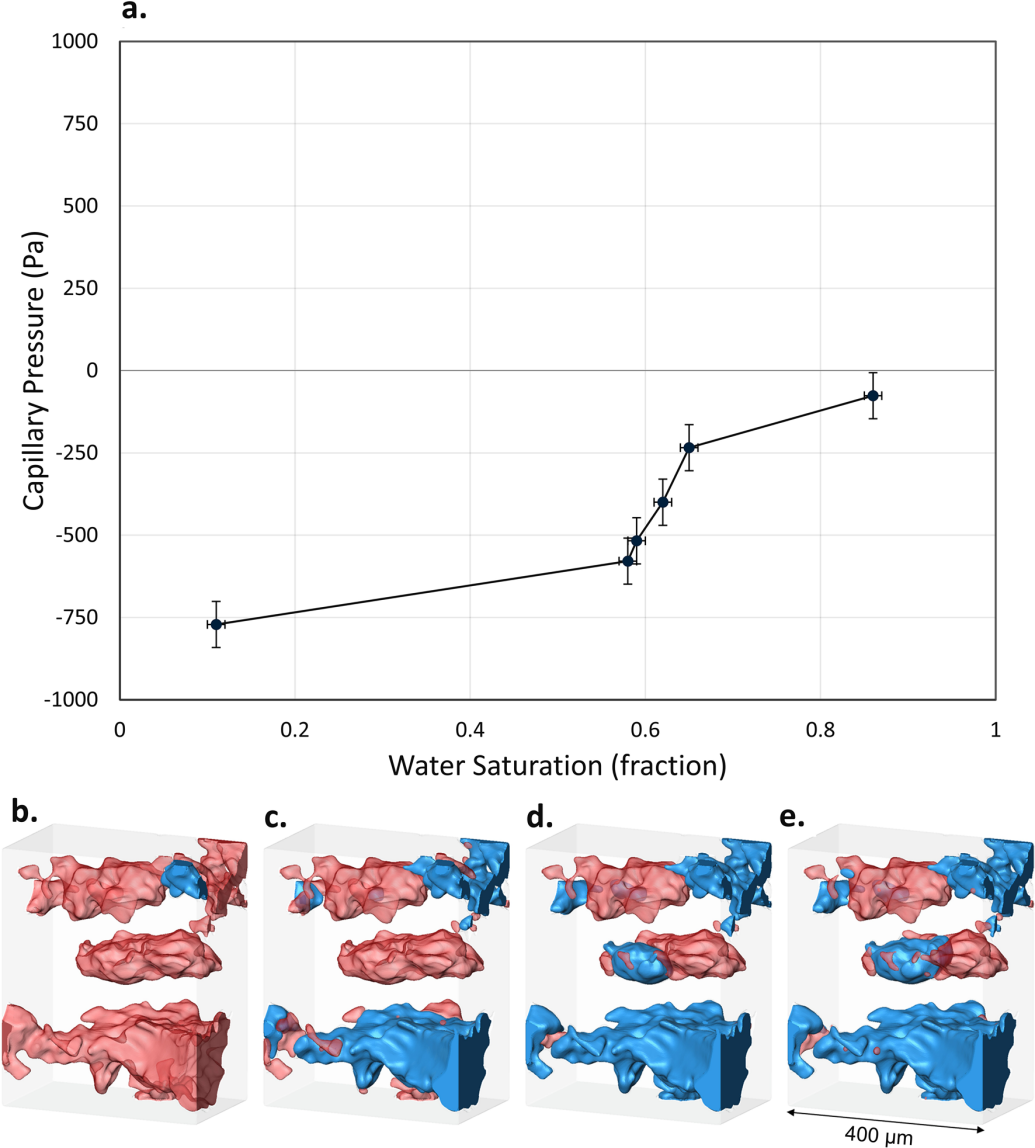
(**a**) The capillary pressure values estimated from the mean interfacial curvature, Fig. 5e, as a function of water (brine) saturation. (**b–e**) A 3D representation of the pore filling sequence during LSW where light grey, red and blue represent the rock, oil and brine phases, respectively. (**b**) Initially, pores were mainly filled with oil. (**c**) When waterflooding started, low salinity brine invaded large pores first with the lowest threshold capillary pressure. (**d,e**) Brine subsequently invaded smaller pores as more pore volumes were injected at higher flow rates. The pores and fluids were visualized using Avizo 9.5 sofware (https://www.fei.com/software/amira-avizo/).

The capillary pressure was calculated from the curvature measurements using the Young–Laplace law:

2 $$\begin{array}{*{20}c} {P_{c} = 2\sigma \kappa } \\ \end{array}$$

where σ is the oil–brine interfacial tension and κ is the mean curvature of the interface. The mean values of the distributions shown in Fig. 5e were used to characterize the capillary pressure of the system^37, 55, 64^ before waterflooding and after each injection step, see Fig. 6a. The negative capillary pressures are consistent with the measured mean curvature values. A strongly negative capillary pressure before waterflooding is a characteristic of predominantly oil-wet media. Figure 6b shows this oil-wet state where a brine ganglion is surrounded by oil in a large pore. Negative pressure values signify a forced displacement where brine must be, on average, at a higher pressure than oil to induce flow over oil-wet surfaces. At this stage, brine can only displace oil from large oil-wet pore elements, Fig. 6c. However, as low salinity brine is injected, capillary pressure values become less negative indicating a lower threshold entry pressure. Only then brine can invade smaller water-wet pores and throats, see Fig. 6d,e. This change in capillary pressure suggests that the rock wettability has been modified after LSW.

Upon initial examination, Fig. 6a appears to show a counter-intuitive, or indeed non-physical, trend of capillary pressure with saturation: normally capillary pressure decreases with increasing water saturation^58^. However, the reason for capillary pressure increasing with saturation is because the wettability changes during the displacement. That is, the normal decreasing trend with increasing saturation is outweighted by the impact of a change in wettability and average interfacial curvature, as shown in Fig. 5e.

**Figure 7:**
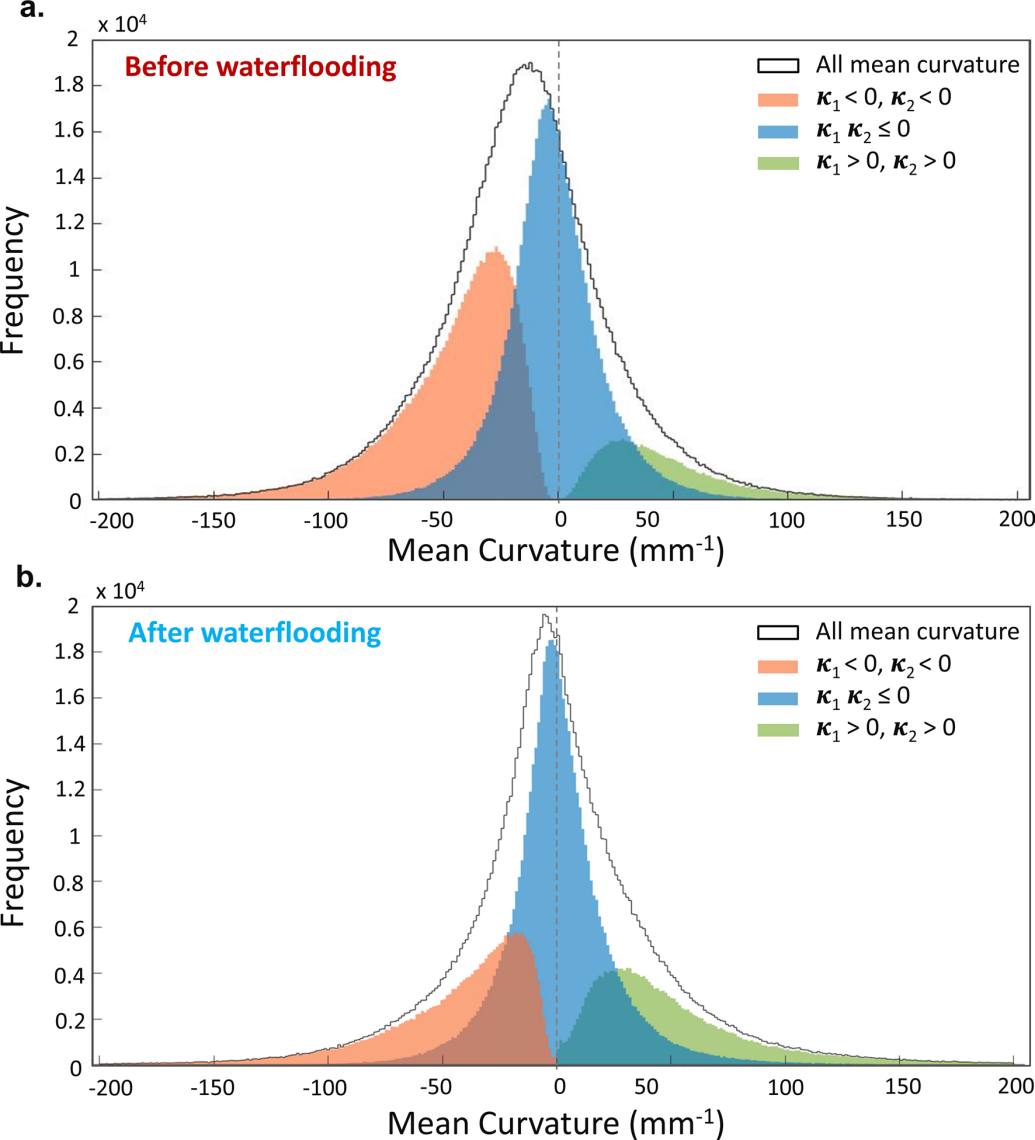
Measured oil–brine curvature distribution before and at the end of waterflooding using the principal curvatures (κ_1_ and κ_2_) obtained from all oil–brine interfaces. The black curve shows the mean curvatures. The distribution of values where principal curvatures are both negative, both positive, and of the opposite signs are shown in orange, green, and blue, respectively. After low salinity waterflooding most of the interfaces have a negative Gaussian curvature.

In addition to examining wettability and capillary pressure, curvatures can be used to evaluate fluid connectivity from the product of the interfacial curvatures in orthogonal directions: the Gaussian curvature^65, 66^*.* In the case of well-connected phases, the Gaussian curvature will be negative, and vice versa. To assess connectivity, principal curvatures were sorted into three categories: κ_1_ < 0, κ_2_ < 0; κ_1_ κ_2_ ≤ 0 and κ_1_ > 0, κ_2_ > 0. Before waterflooding, there is a broader distribution with primarily negative principal curvatures, Fig. 7a. When the interfaces have two negative principal curvatures, i.e. positive Gaussian curvature, this indicates an oil-wet system with the water phase poorly connected in the pore space^64^; an opposite of what is observed in water-wet cases^36^. After LSW, the majority of the curvatures have one positive and one negative value, i.e. a negative Gaussian curvature, Fig. 7b. This implies a mixed-wet system with good connectivity of the phases in the pore space^36, 37, 64^ which results in enhanced oil flow and better recovery.

## Conclusions

This experimental study provides novel insights into secondary LSW through quantitive characterization of wettability alteration associated with the low salinity effect using X-ray micro-CT images of coreflooding of an aged carbonate rock at subsurface conditions. While the experiment was conducted on only a single sample, we suggest that the findings are applicable to LSW in most carbonate rocks. In situ wettability was characterized by (i) pore occupancy mapping and filling order, (ii) contact angle and curvature measurements, and (iii) capillary pressure calculations. We also related these finding to fluid saturation and oil recovery.

The oil-wet nature of the sample, after ageing, was confirmed by the high contact angle, 124°, as well as the negative mean curvature and capillary pressure values. Spatial analysis of fluid occupancy showed the effects of LSW on pore-filling events. Brine, as the non-wetting phase, initially invaded large pores. However, as the waterflood continued, brine progressively invaded smaller pores and oil was displaced into larger pores. This is the first time this pattern of pore-filing and oil re-distribution is observed and it indicates a gradual wettability alteration in the sample by LSW. This was further proved by an observed shift in contact angle and mean curvature distribution towards an average contact angle of 108° and a mean curvature close to zero. The calculated capillary pressure values are consistent with a change from an oil-wet system towards a mixed-wet system, with a capillary pressure close to zero at the final water saturation.

After the injection of 60 pore volumes at different flow rates, a prolonged response to low salinity injection is showcased in this study. The changes in the wettability configuration in the system and the good connectivity of the oil phase were the main factors responsible for a remarkable recovery performance and a low residual oil saturation, 15%, in the resolved porosity.

This work provides hitherto unreported insights into pore-scale displacements under LSW and can help interpret results obtained from core-scale experiments. The results offer a valuable benchmark for pore-scale modelling and upscaling to help design LSW EOR projects at the field scale. Further work could compare the pore-scale displacement observed here with the results from a tertiary LSW. Moreover, the same methodology for wettability characterisation on a pore-by-pore basis can be applied to study displacement in oil- and mixed-wet porous media in other applications, such as carbon storage, flow in batteries and packed bed chemical reactors.

## Supplementary Information

Supplementary Information.
