# Supplementary material for: Pore-scale imaging and analysis of low salinity waterflooding in a heterogeneous carbonate rock at reservoir conditions
Source: Sci Rep. 2021 Jul 23;11:15063. doi: 10.1038/s41598-021-94103-w (PMC8302661; doi:10.1038/s41598-021-94103-w)
Supplement: Supplementary file 1 — Supplementary Information. [file 41598_2021_94103_MOESM1_ESM.pdf]

Supplementary Information

Pore-scale imaging and analysis of low salinity waterflooding in a heterogeneous carbonate rock at reservoir conditions

Ahmed M. Selem [a*], Nicolas Agenet [b], Ying Gao[a], Ali Q. Raeini[a], Martin J. Blunt[a] & Branko Bijeljic[a]

[a] Department of Earth Science and Engineering, Imperial College London, London, United Kingdom

[b] TOTAL E&P France, Pau, France

* a.selem18@imperial.ac.uk

Rock sample

The rock sample used in this study was Estaillades limestone, a quarry carbonate from France, which has a heterogeneous pore structure similar to that found in carbonate reservoir rocks. Estaillades has a bimodal pore size distribution; prolific intergranular macro-porosity and abundant micro-porosity contained in the bioclasts. A cylindrical sample, 5.9 mm in diameter and 10 mm in length, was drilled out of a core plug, 5 cm in diameter and 10 cm in length, with a mechanical drilling machine.

Flow apparatus, experimental procedure, and ageing protocol

The experimental apparatus is made up of a high-pressure high-temperature flow cell connected, via PEEK tubing and valves, to oil injection, brine injection, receiving and confining syringe pumps (Fig. S1). The Teledyne Isco pumps were used to tightly confine the sample and make a pressure difference to control the rate of fluids flow inside the sample. The sample was placed inside a rubber Viton sleeve to isolate the rock and injected fluids from the confining fluid. Metal end pieces, connected to injection and receiving lines, were attached to the inlet and outlet of the sleeve.

The experimental procedure consisted of the following steps:

1. A pressure of 2 MPa using deionized water was applied in the confining isolated space between the Viton sleeve and the carbon fibre sleeve and a dry (air) scan of the sample was taken.
2. The sample was initially flooded with brine solution made from deionized water with 20 weight% potassium iodide (KI). This brine can be distinguished from rock phases for effective image segmentation and characterization of the rock bimodal porosity[1].
3. The sample was then cleaned with four-times diluted formation brine followed by isopropanol both injected for 10 pore volumes. It was dried with a gentle nitrogen

flux for 24 hours then vacuumed for 3 hours. Formation brine was then injected to fully saturate the rock sample.

4. Drainage was performed with the injection of viscous synthetic oil to reach irreducible water saturation. This oil has a relative density and dynamic viscosity (measured at 20 °C) of 836 kg/m^3 and 12 mPa.s respectively.
5. Toluene was then injected for 10 PVs followed by the injection of crude oil at an increased temperature, 50 °C to avoid asphaltene precipitation.
6. The sample was then aged with a continuous injection of crude oil for three weeks at a low flow rate (2 μL/min) at high pressure (11 MPa) and temperature (80 °C) to change the rock wettability to a similar state found in oil reservoirs. The flow direction was reversed at mid-point during ageing.
7. After ageing, the crude oil was doped with 20 weight% 1-iododecane as a high contrast dopant to distinguish the oil phase from the brine and rock phases in the X-ray images.
8. The temperature in the system was then changed to 70 °C using an Omega flexible heater and a PID controller as shown in Fig. S1.
9. Low salinity brine was then injected at a sequence of six waterfloods. At each step 10 pore volumes were injected at a different flow rate. The flow rates and corresponding capillary number are provided in Table S1.

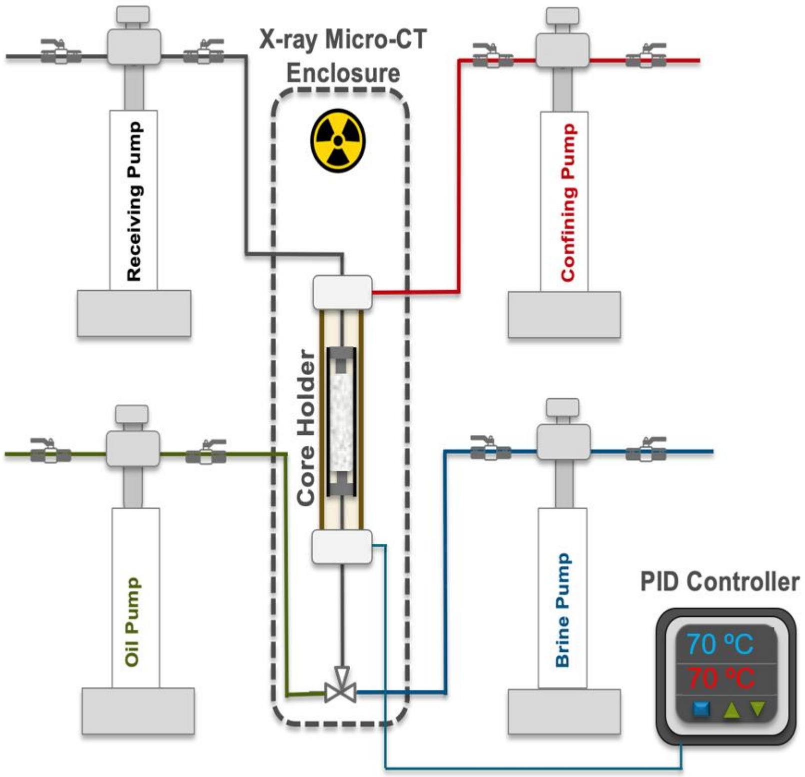

Figure S1. The experimental flow apparatus consisted of core holder assembly, syringe pumps to apply back pressure and flow rates and a PID controller to apply temperature on the rock and fluids inside the micro-CT.

Table S1. Low salinity waterflooding injection steps with calculated capillary numbers. A total of 60 pore volumes were injected throughout waterflooding with 10 pore volumes at each rate.

Injection step (Image name)	Flow rate (μL/min)	Pore volume injected (PVI)	Capillary number (Ca)
LSW1	1	10	9.72×10^{-9}
LSW2	2	10	1.94×10^{-8}
LSW4	4	10	3.89×10^{-8}
LSW11	11	10	1.07×10^{-7}
LSW22	22	10	2.14×10^{-7}
LSW42	42	10	4.08×10^{-7}

Image acquisition, processing, and segmentation

Image acquisition was performed with 95 keV energy, exposure time of 0.8 s and 3600 projections with the continuous helical movement of the sample and 360° rotation. The resultant 3D-images (tomograms) were registered, filtered and segmented, then used for fluid saturation and pore occupancy analyses. A smaller sub-volume was extracted for the contact angle, curvature, and capillary pressure analyses, Fig. S2.

The images were filtered using non-local means edge-preserving filter to eliminate image noise[2]. A watershed algorithm was used for image segmentation. This algorithm is based on generating a seed using a two-dimensional histogram of both the greyscale and the greyscale gradient images[3,4], as shown in Fig. S3. A mask of segmented pores from the dry scan was applied on all subsequent images to separate the macro-pores from the rest of the rock and to simplify the segmentation of the oil and brine within, Fig. S4.

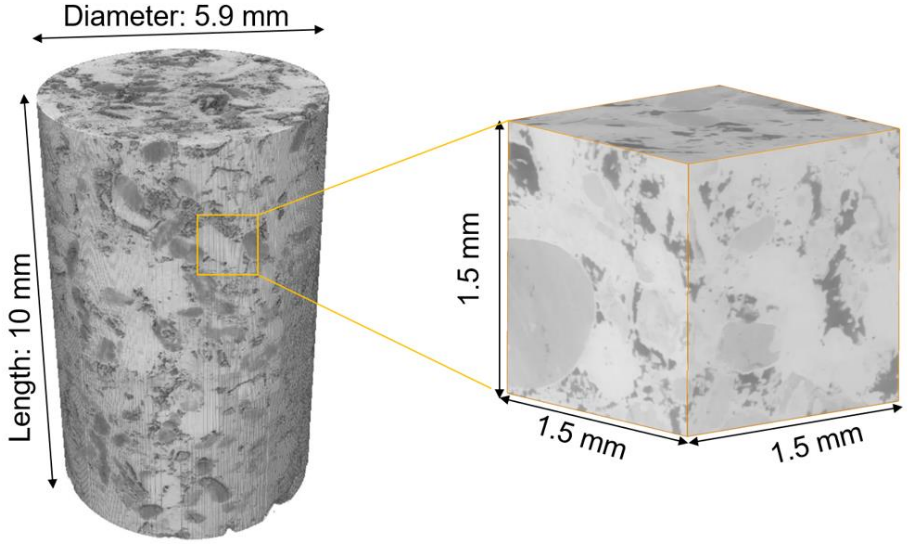

Figure S2. A tomogram of the sample used in this study with a total volume of 28.8 billion voxels. Contact angle and curvature measurements were performed on an extracted sub-volume (1.5 mm^3)

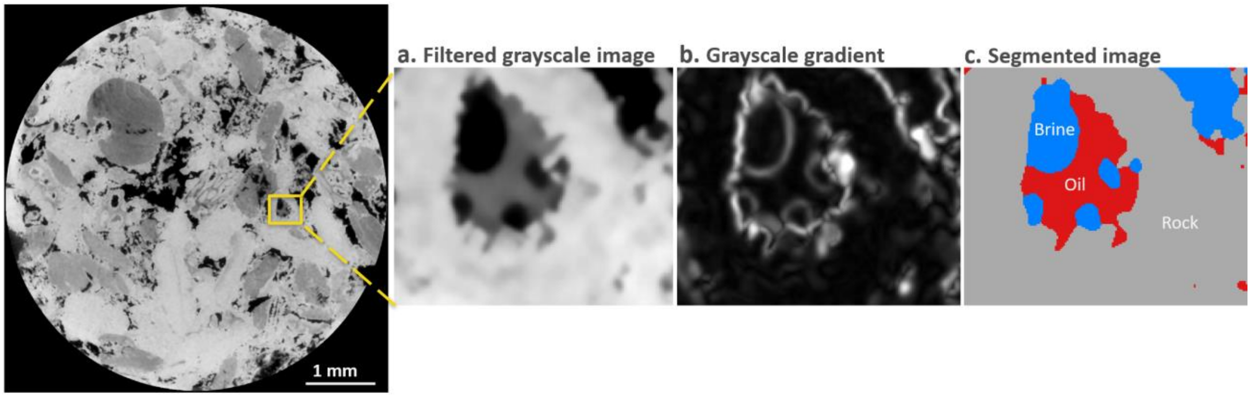

Figure S3. An example showing the segmentation workflow using seeded watershed algorithm. A seed is generated using a two-dimensional histogram of both the greyscale (a) and the greyscale gradient (b) images to produce a labelled image (c) showing the different phases: brine, oil and rock.

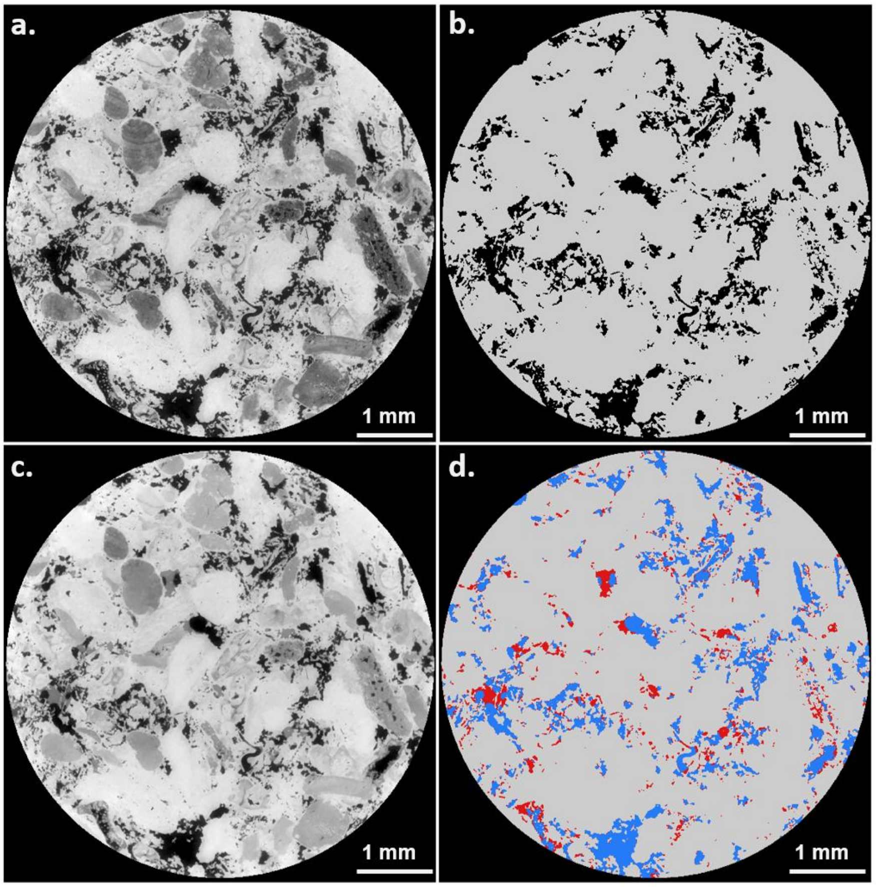

Figure S4. Image segmentation workflow. A filtered two-dimensional cross-section of the three-dimensional micro-CT dry image (a), which is segmented into resolved pores (black) and grains (grey) in (b). An orthogonal slice from an image of the sample saturated with brine and doped oil at the end of low salinity waterflooding (c). The pores mask was applied to the saturated sample image to separate the pore space and segment it into oil and brine shown in red and blue respectively, and then the rock phase in grey was added (d).

Pore space characterization (differential imaging)

To accurately quantify sub-resolution porosity, a technique called differential imaging is used[1]. A brine solution made from de-ionized water doped with 20 weight% Potassium Iodide (KI), as a high contrast agent, was used in the characterization of sub-micron porosity. This dense brine can be distinguished from rock phases for effective image segmentation and characterization of the rock bimodal porosity. As mentioned previously in the experimental procedure steps, X-ray micro-CT was used to acquire a dry (air) image of the rock and an image after KI-brine injection. To maximize the phase contrast between the grain and pore phases, a differential image between the KI-saturated and dry images was obtained. This image was then segmented using the seeded watershed method into solid grains, sub-resolution pores and macro pores. This differential imaging workflow is shown in Fig. S5. The segmented label images combined with the KI-saturated image were used to obtain histograms of the grey-scale voxels for each phase (Fig. S6). The peak values (CT) of the histograms were used to calculate the micro-porosity fraction (ϕ_{micro}),

$$\phi_{\mathrm{micro}} = \frac{CT_{\mathrm{micro}} - CT_{\mathrm{grain}}}{CT_{\mathrm{macro}} - CT_{\mathrm{grain}}} \tag{1}$$

The total pore space comprises the macro- and macro-pores,

$$\phi_{\mathrm{total}} = \phi_{\mathrm{micro}} \times V_{\mathrm{micro}} + 1 \times V_{\mathrm{macro}} \tag{2}$$

where V_{micro} and V_{macro} are the total volume fractions for each phase obtained from the segmented image. The total porosity from differential imaging was 0.299 compared to a value of 0.293 measured by a Helium porosimeter (measurement conducted at Imperial College London). A detailed explanation of the differential imaging method can be found in ref. 1.

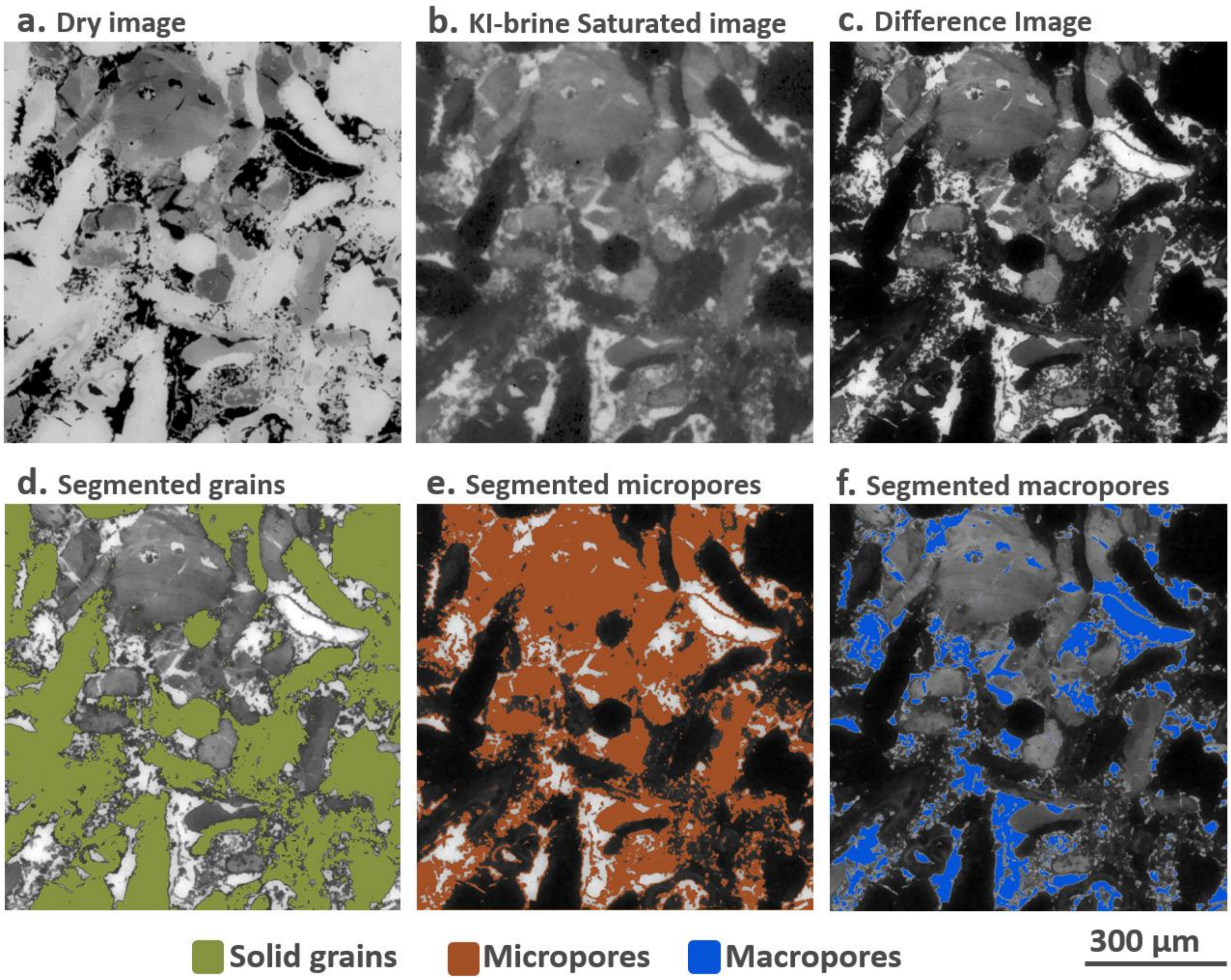

Figure S5. Two-dimensional cross-sections of three-dimensional micro-CT images at the same location in the Estaillades sample. (a) The dry scan. Air in the pore space does not adsorb X-rays and is black in the image. (b) The brine-saturated image. Brine has a higher X-ray attenuation than solid and appears bright (white) in the image. (c) The difference image between (b) and (a). The black in (c) represents the impermeable solid grains, white is macro pore space, while intermediate grey values indicate micro-porosity where the pore structure cannot be explicitly resolved in the image. Segmentation classification shown as two-dimensional cross-sections of the three-dimensional images of the same slice: (d) Solid grains (green). (e) Micro-pore phase (orange). (f) Macro-pore space (blue).

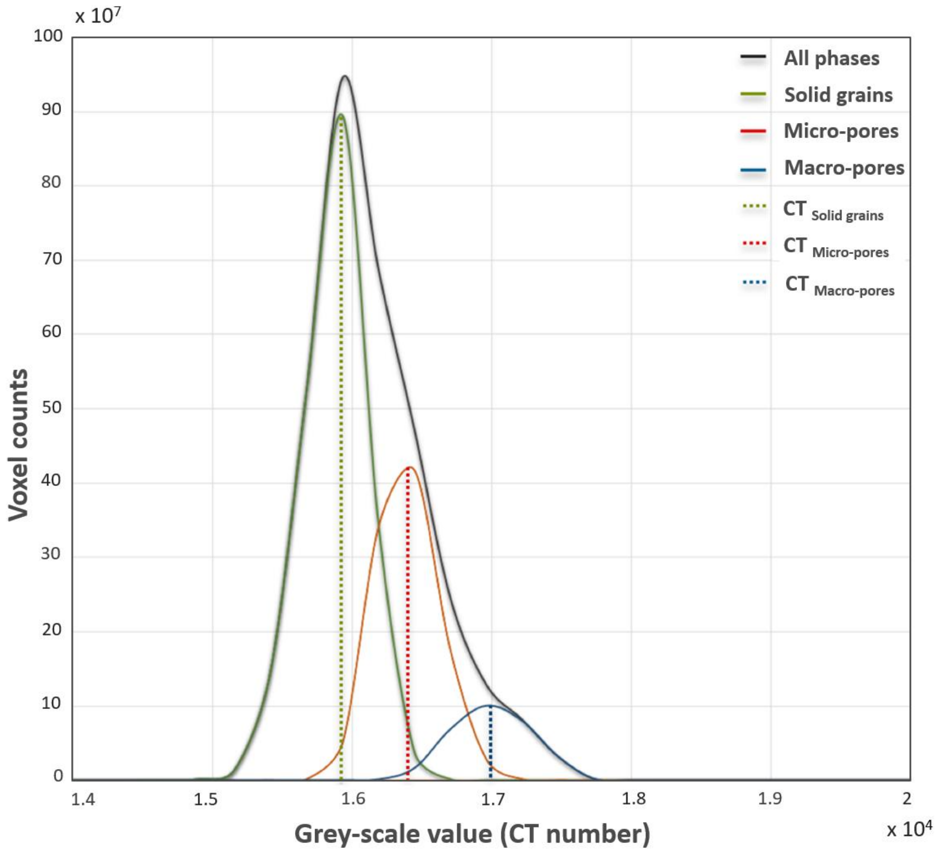

Figure S6. Histograms of the grey-scale values of the phases in the KI saturated image (Fig. S5b) used to calculate the fraction of unresolved pores in the micro-porous rock grains.

Image analysis

Pore occupancy analysis

The distribution of pore elements fluid occupancy, sizes and location were investigated using a generalized pore network extraction algorithm[5]. The algorithm divided the pore space into pores (wider regions) connected with throats (narrower regions) with voxels assigned to unique pores and throats (Fig. S7a). The volume-weighted fractions of pore elements whose centers were occupied mainly by either brine or oil were calculated to assess the fluid occupancy at different stages before and during low salinity waterflooding (Fig S7b-d).

Contact angle measurements

Contact angle measurements were conducted on the segmented images of a 1.5 mm × 1.5 mm × 1.5 mm sub-volume using an automated algorithm[6]. The number of contact points and values of contact angles measured before and during waterflooding is shown in Table. S2.

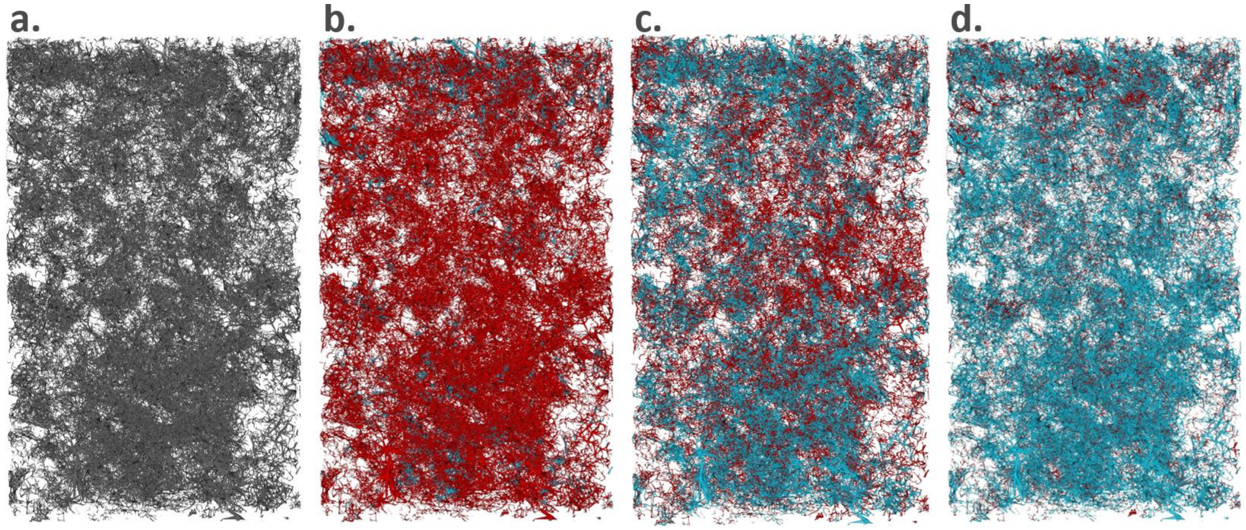

Figure S7. Pore network extraction and fluid occupancy mapping from the 3D segmented images. (a) A pore network extracted from the segmented dry image of the whole sample with 5.9 mm diameter and 10 mm length. (b-d) Occupancy maps of oil (red) and brine (blue) before, during and at the end of low salinity water injection, respectively.

Table S2. Contact angles statistics and results. The measurements were conducted on a sub-volume with a total volume of 1.5 mm3 (132.8 million voxels).

**Injection rate** (μL/min)	**Counts** (thousands)	**Mean contact angle** (°)	**Standard deviation** (± °)
Before waterflooding (after ageing)	54.13	124.0	22.9
1	201.64	114.3	24.1
2	190.17	114.1	22.3
4	154.08	113.5	24.7
11	142.07	113.6	20.3
42	106.34	107.7	22.8

Curvature measurements

The curvatures were analyzed from the fluid interfaces extracted from the same sub-volume on which contact angles measurements were performed. The voxelized interfaces were smoothed using a volume preserving Gaussian smoothing[7], Fig. S8. The mean of the two principal curvatures (κ_1 and κ_2) was calculated as $\kappa = (\kappa_1 + \kappa_2)/2$.

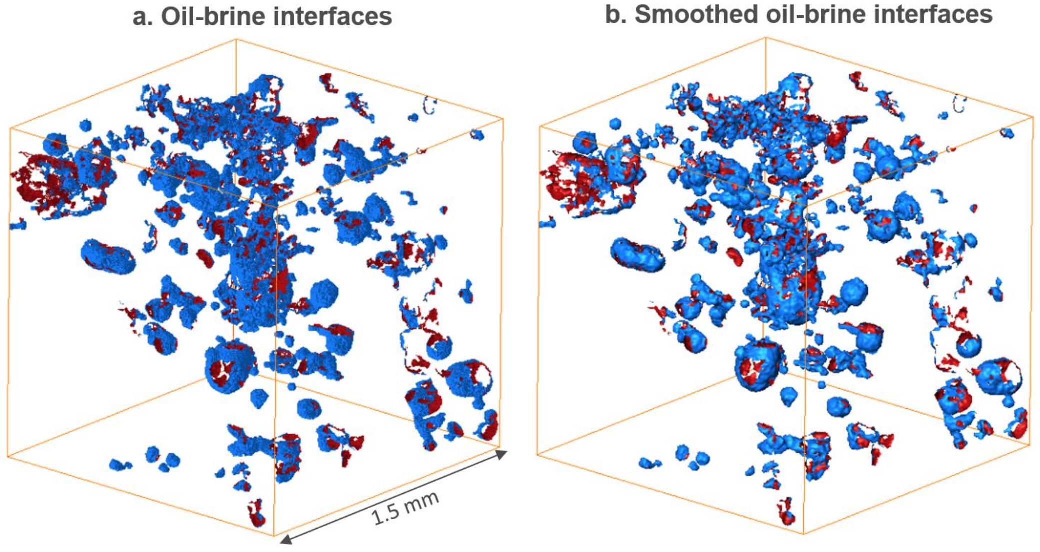

Figure S8. Oil-brine interfaces were smoothed using volume preserving Gaussian smoothing (kernel size 5) to remove voxelization artefacts. Curvatures were measured on the smoothed interfaces. The interfaces were extracted using Avizo 9.5.

References

1 Lin, Q., Al-Khulaifi, Y., Blunt, M. J. & Bijeljic, B. Quantification of sub-resolution porosity in carbonate rocks by applying high-salinity contrast brine using X-ray microtomography differential imaging. *Advances in Water Resources* **96**, 306-322, doi:https://doi.org/10.1016/j.advwatres.2016.08.002 (2016).

2 Buades, A., Coll, B. & Morel, J. in *2005 IEEE Computer Society Conference on Computer Vision and Pattern Recognition (CVPR'05).* 60-65 vol. 62.

3 Jones, A. C. *et al.* Assessment of bone ingrowth into porous biomaterials using MICRO-CT. *Biomaterials* **28**, 2491-2504 (2007).

4 Andrew, M., Bijeljic, B. & Blunt, M. J. Pore-scale imaging of trapped supercritical carbon dioxide in sandstones and carbonates. *International Journal of Greenhouse Gas Control* **22**, 1-14, doi:https://doi.org/10.1016/j.ijggc.2013.12.018 (2014).

5 Raeini, A. Q., Bijeljic, B. & Blunt, M. J. Generalized network modeling: Network extraction as a coarse-scale discretization of the void space of porous media. *Physical Review E* **96**, 013312 (2017).

6 AlRatrout, A., Raeini, A. Q., Bijeljic, B. & Blunt, M. J. Automatic measurement of contact angle in pore-space images. *Advances in Water Resources* **109**, 158-169, doi:https://doi.org/10.1016/j.advwatres.2017.07.018 (2017).

7 Taubin, G. in *Proceedings of IEEE international conference on computer vision.* 852-857 (IEEE).
